# Oxidative status predicts quality in human mesenchymal stem cells

**DOI:** 10.1186/s13287-016-0452-7

**Published:** 2017-01-06

**Authors:** Alessandro Bertolo, Simona Capossela, Gion Fränkl, Martin Baur, Tobias Pötzel, Jivko Stoyanov

**Affiliations:** 1 Biomedical laboratories, Swiss Paraplegic Research, Nottwil, Switzerland; 2 Cantonal Hospital of Lucerne, Lucerne, Switzerland; 3 Swiss Paraplegic Centre, Nottwil, Switzerland; 4 Institute for Surgical Technology and Biomechanics, University of Bern, Bern, Switzerland

**Keywords:** Human mesenchymal stem cells, Cell senescence, Cell differentiation, Antioxidant enzymes, Mitochondrial biogenesis, Methylene blue

## Abstract

**Background:**

Human bone marrow-derived mesenchymal stem cells (MSC) are adult progenitor cells with great potential for application in cell-based therapies. From a cell-based therapy perspective, there are two limitations to MSC use: (1) these therapies require large numbers of cells, and long-term expansion of MSC in vitro promotes replicative senescence; and (2) patient variability is a challenge for defining MSC quality standards for transplantation. This study aimed to determine whether low or high oxidative status of MSC correlate with changes in cell expansion and differentiation potentials.

**Methods:**

We investigated functional aspects of mitochondria, such as cell metabolic activity indicators and expression of antioxidant enzymes. Furthermore, we tested if senescence-induced changes in oxidative status of MSC could be counteracted by methylene blue (MB), an alternative mitochondrial electron transfer known to enhance cell bioenergetics.

**Results:**

MSC isolated from donors of the same age showed distinctive behavior in culture and were grouped as weak (low colony-forming units (CFU) and a short life in vitro) and vigorous MSC (high CFU and a long life in vitro). In comparison to weak MSC, vigorous MSC had oxidative status characterized by lower mitochondrial membrane potential, lower mitochondrial activity, and fewer reactive oxygen species production, as well as reduced mitochondrial biogenesis. Vigorous MSC had a significantly higher expansion potential compared to weak MSC, while no differences were observed during differentiation. MB treatment significantly improved expansion and differentiation potential, however only in vigorous MSC.

**Conclusions:**

Together, these results demonstrate the importance of mitochondrial function in MSC in vitro, and that cells with low oxidative status levels are better candidates for cell-based therapies.

## Background

Mesenchymal stem cells (MSC) are adult progenitor cells associated with the musculoskeletal lineage, and are characterized by fast expansion ability [1], differentiation into several mesodermal phenotypes [2], and secretion of trophic and immunomodulatory factors [3]. MSC can be isolated from diverse adult tissues, including bone marrow stroma [4]. The number of MSC in the bone marrow might be reduced by diseases such as osteoporosis [5], leukemia [6], or lung and breast cancers [7]. Aging has also been proposed as an underlying cause of MSC deficiency in the bone marrow; however, this is still a matter of debate. While a decline in MSC number in bone marrow stroma has been linked to increased donor age in several papers [8, 9], others report the absence of any correlation between the two factors [6, 10]. The reasons for exhaustion of MSC in the bone marrow are still debated; however, oxidative stress has been shown to be one of the major factors influencing tissue aging [11, 12], and as such merits in-depth investigation.

Oxidative stress exists in every cell, but as a pathological condition it is based on the disruption of the physiological equilibrium between oxidant and antioxidant species, which leads to abnormal intracellular accumulation of reactive oxidative species (ROS). ROS—such as superoxide radicals and hydrogen peroxide—has a detrimental impact on biomolecules, cells, and tissues [13], contributing over time to the development of some of the more common diseases occurring with aging, such as cardiovascular disease, diabetes, and cancer [14]. In a healthy homeostasis, ROS are balanced by the action of antioxidant enzymes, the expression of which declines with aging [15]. Furthermore, young and functional cells maintain a delicate equilibrium between fission and fusion of mitochondria [16], and defective mitochondrial turnover and dynamics has been implemented in the process of aging [17]. Equilibrium is also fundamental at the molecular level in the mitochondria, especially between nuclear and mitochondrial encoded proteins. Stoichiometric imbalance in these proteins leads to the activation of the mitochondrial unfolded protein response (UPR^mt^) which protects mitochondria from further stress and promotes prolongation of the lifespan [18]. Lifespan extension is also related to increased levels of an electron carrier, the metabolic cofactor NAD^+^ [19, 20]. NAD^+^ supports the deacetylation activity of sirtuin-1 (SIRT1) which promotes the expression of genes involved in mitochondrial biogenesis, such as peroxisome proliferator-activated receptor gamma coactivator 1-alpha (PGC-1α) [21]. Since activation of SIRT1 depends on NAD^+^ levels, increasing its levels or supplying an additional electron donor could counteract mitochondrial dysfunction.

Methylene blue (MB), a diaminophenothiazine, is a functional electron donor due to its low redox potential [22], and the activity of MB within the mitochondria depends on NAD(P)H dehydrogenases resulting in the formation of its reduced form, MBH_2_ (leucomethylene blue) [23]. It acts as a substitute electron carrier that can bypass the compromised electron transport chain at proximal complexes, interacting with complex I and cytochrome c [24]. The beneficial role of MB was demonstrated in a mouse model of Hutchinson-Gilford progeria—a premature aging syndrome—where MB rescued damaged mitochondria and prolonged the in vitro lifespan of cell cultures to levels similar to that of the wild-type control [25].

Here, we hypothesized that the structure and function of mitochondria degenerate during the process of in vitro aging of human MSC, and our aim was to correlate the oxidative status of MSC to their expansion potential. For this purpose, MSC were split into weak and vigorous cultures based on their in vitro fitness. Then, we investigated some structural and functional features of mitochondria during cellular aging, including cell metabolic activity indicators and antioxidant enzyme protein expression. We also tested whether degenerative changes in the oxidative status could be counteracted by supplementation of MB. Finally, we investigated the impact of MB on MSC differentiation potential to determine if there is a correlation between mitochondrial dysfunction and differentiation to adipogenic, chondrogenic, and osteogenic lineages, assessed by quantification of tissue-specific markers.

## Methods

### MSC isolation and expansion

Fresh bone marrow (BM) samples were obtained from the iliac crest of the donors during surgery. MSC were isolated from BM of six male donors (average age 65 ± 8 years; *n* = 3 per group). The BM aspirates were diluted in 3.8% sodium citrate and phosphate-buffered saline (PBS; Applichem, Axonlab, Baden, Switzerland) and then filtered through a 100-μm cell strainer to remove clots (Falcon, Faust, Schaffhausen, Switzerland). Mononuclear cells were separated by H-Lympholyte Cell Separation Media gradient centrifugation (density 1.077 g/mL; Cedarlane, Bio Concept, Allschwil, Switzerland) in a Leucosep tube (Huberlab, Reinach, Switzerland) at 800 *g* for 15 min, washed with PBS, centrifuged again at 210 *g* for 10 min and plated at a density of 1 × 10^5^ cells/cm^2^ in tissue culture flasks (TPP, Faust) in α-MEM, supplemented with 10% fetal bovine serum (FBS) (both Amimed, Bio Concept), 100 units/mL penicillin and 100 mg/mL streptomycin, and 2.5 μg/ml amphotericin B (both Gibco, LuBioScience GmbH, Lucerne, Switzerland) at 37 °C in a humid atmosphere containing 5% CO_2_. After 2 days, non-adherent cells were discarded, whereas adherent cells were cultured in growing medium consisting of DMEM/Ham’s F12, supplemented with 10% FBS (both Amimed), 100 units/mL penicillin and 100 mg/mL streptomycin, 2.5 μg/ml amphotericin B (all Gibco) and 5 ng/ml recombinant basic fibroblast growth factor (bFGF; Peprotech, LuBioScience), with medium changed three times a week.

### Colony forming assay

Freshly isolated cells from bone marrow (mononuclear cells; 1 × 10^6^) were plated in 10-cm Primaria cell-culture dishes (Falcon) and cultured with growing medium. After 14 days, cell colonies were washed with PBS, fixed with 100% methanol, and stained with Giemsa solution (all Applichem).

### Long-term culture of MSC

MSC plated at a density of 1.6 × 10^3^ cells/cm^2^ were cultured with or without the addition of 200 nM MB (Applichem) to the growing medium. Cultures were split at 80–90% confluency and re-plated at the same cell density. Cell number and cell diameter were measured with the Scepter cell counter (Millipore, Milian, Nesselnbach, Switzerland) at each passage. The population doublings per passage (PDP) were calculated by the formula PDP = ln(n_f_/n_i_), where n_i_ is the initial number of cells and n_f_ the final number of cells. The division rate for each passage was calculated by dividing PDP by time. For each donor, results per passage were pooled in three equal groups, namely early, middle, and late passages (minimum 3 and maximum 6 passages per group depending on how long the cell culture protracted).

### Flow cytometry analysis of MSC markers

MSCs were sampled at 1 × 10^5^ cells/tube to investigate the proportion of CD44-, CD90-, and CD105-positive and CD14-negative cells. Cells were incubated with CD14-FITC (NB100-77759, Novus Biological, LuBioScience), CD44-FITC (NBP1-41278, Novus Biological), CD90-FITC (NBP1-96125, Novus Biological), and CD105-FITC (MCA1557A488T, AbD Serotec, LuBioScience) antibodies in PBS for 1 h at 4 °C, washed and resuspended in PBS. Cell fluorescence was evaluated with FACScalibur flow cytometer (Becton Dickinson) and data were analyzed using FlowJo v.10.0 software (Treestar, Ashland, OR, USA).

### Senescence associated beta-galactosidase assay (SA-β-Gal)

SA-β-gal activity of MSC was determined at different passages using a fluorescence-based method and flow cytometry, as described previously [26, 27]. In brief, cells were pre-treated for 1 h with 100 nM bafilomycin A1 (Sigma, Buchs, Switzerland), which inhibited lysosomal acidification, and then incubated for 1 h with 33 μM 5-dodecanoylaminofluorescein di-β-D-galactopyranoside (C_12_FDG, Sigma), a fluorogenic substrate for β-galactosidase. Data were analyzed with FlowJo software.

### Cell metabolic activity

At each passage, cell activity was assessed by resazurin reduction assay. MSC were incubated in resazurin solution (15 ng/mL resazurin, 2.5 ng/mL methylene blue, 0.1 mM potassium ferricyanide, 0.1 mM potassium ferrocyanide (all Applichem) in growing medium without bFGF) for 3 h and bottom well fluorescence absorbance was measured (λ_ex_ = 535 nm and λ_em_ = 595 nm) using a Multimode Detector (DTX 880; Beckman Coulter, Nyon, Switzerland). Results were normalized to the amount of cells.

### Mitochondrial membrane potential and superoxide mitochondrial accumulation

MSC were tested with the JC1-Mitochondrial Membrane Potential Assay Kit (Abnova, LuBioScience) which contains tetraethylbenzimidazolylcarbocyanine iodide (JC-1; LucernaChem, Lucerne, Switzerland), a cationic dye that accumulates in energized mitochondria. At low mitochondrial membrane potential JC-1 is predominantly a monomer (λ_ex_ = 535 nm), while at high mitochondrial membrane potential the dye aggregates (λ_ex_ = 595 nm). Cells were incubated for 15 min with JC-1 in growing medium, followed by three washes with PBS. Mitochondrial membrane potential was calculated as the ratio between JC-1 aggregate and monomer.

The production of superoxide by mitochondria was measured using the MitoSOX Red reagent (Thermo Fisher, Zug, Switzerland). Cells were incubated for 10 min with 5 μM MitoSOX Red and 1× SYBR (Thermo Fisher) in HBSS (Gibco), followed by three washes with HBSS. The oxidized product is highly fluorescent upon binding to nucleic acid. Bottom well fluorescence absorbance was measured using a Multimode Detector for both methods and results were normalized on RFU of SYBR.

### RNA isolation, cDNA synthesis, and real-time PCR

Total RNA was isolated from MSC microcarrier constructs after 28 days in culture. Constructs were homogenized using Dispomix in RNA lysis buffer of Aurum Total Mini Kit (Bio Rad, Reinach, Switzerland), following the manufacturer’s instructions with the modification of adding 2 μl polyacryl carrier (LucernaChem) in the kit lysis buffer. cDNA was prepared using VILO cDNA Synthesis Kit (Invitrogen).

Real-time (RT)-PCR reactions consisted of the primers listed in Table 1 at a concentration of 250 nM, 5 μl cDNA template, and IQ SYBR Green Supermix (Bio Rad). Specific products were amplified by a quantitative PCR system (CFX96™ Real Time System, Bio Rad). RT-PCR was carried out with the following settings: denaturation 95 °C 3 min (1 cycle), 95 °C 15 s, 60 °C 20 s, and 72 °C 20 s (35 amplification cycles) in a final volume of 20 μl in 96-well plates (Bio Rad). Melting curve analysis was performed after each reaction. Relative gene expression was determined using the 2^−ΔΔCt^ method and the results were normalized to the expression of GAPDH.

**Table 1:** Human genes used in quantitative real-time PCR

Gene	Primer nucleotide sequence (5′ → 3′)	Amplicon (bp)
Housekeeping gene		
GAPDH	F - TGGACTCCACGACGTACTCA	102
	R - GGAAGCTTGTCATCAATGGAA	
Mitochondrial biogenesis		
POLG	F - GCTGGTGGAAGAGCGTTACTC	241
	R - GAAGCTGCTTAGCCCTGAGAT	
POLG2	F - GGTTTGGGGGTCGAGTAGATG	186
	R - TTCCACTTAGGAAATGCCTTCTC	
NRF1	F - AACAAAATTGGGCCACGTTACA	291
	R - TCTGGACCAGGCCATTAGCA	
TFAM	F - ATGGCGTTTCTCCGAAGCAT	133
	R - CAGATGAAAACCACCTCGGTAA	
PGC-1α	F - GCTTTCTGGGTGGACTCAAGT	142
	R - TCTAGTGTCTCTGTGAGGACTG	
PGC-1β	F - CCACATCCTACCCAACATCAAG	93
	R - CACAAGGCCGTTGACTTTTAGA	
Mitochondrial chaperones		
PHB	F - CACAGAAGCGGTGGAAGC	96
	R - ATGGCCGCCTITTTCTGT	
PHB2	F - TGCTGAACCTACAGGATGAAA	77
	R - TGGTGACTAGGCTCATITCTTACC	
MRP5S	F - GAAATCGATCCGTGTCTTGG	87
	R - GCATCCATCCGATCAGTAGC	
Telomere length		
Telomere	teloF - CGGTTTGTTTGGGTTTGGGTTTGGGTTTGGGTTTGGGTT	>76
	teloR - GGCTTGCCTTACCCTTACCCTTACCCTTACCCTTACCCT	
36B4	F - CAGCAAGTGGGAAGGTGTAATCC	75
	R - CCCATTCTATCATCAACGGGTACAA	
mtDNA copy number		
ND1	F - GGAGTAATCCAGGTCGGT	265
	R - TGGGTACAATGAGGAGTAGG	
CF	F - AGCAGAGTACCTGAAACAGGAA	482
	R - AGCTTACCCATAGAGGAAACATAA	

*bp* base pair, *F* Forward, *mtDNA* mitochondrial DNA, *R* Reverse

### DNA isolation and determination of telomere length and mtDNA copy number

Telomere length was determined by real-time PCR, as previously described [28]. Briefly, genomic DNA (from 1 × 10^6^ cells) of MSC at early, middle, and late passages was isolated using the appropriate DNA purification kit [29] (Gentra PureGene Cell Kit; Qiagen, Hombrechtikon, Switzerland). gDNA samples were quantified and run in triplicate in a 96-well plate in a quantitative PCR system. The sequences of the telomere and 36B4 primers are listed in Table 1. The real-time PCR program consisted of initial denaturation at 95 °C for 5 min, followed by 32 PCR cycles at 95 °C for 10 s and 60 °C for 30 s. Melting curve analysis was carried out for each reaction and standard curves were fitted for both telomere DNA and 36B4 DNA. For each sample, values were calculated as a ratio of telomere DNA to 36B4 DNA, and data were converted to base pairs based on standard curves of appropriate oligomers.

Mitochondrial DNA (mtDNA) copy number, relative to the diploid chromosomal DNA content, was quantified using a quantitative PCR system [30]. The relative mtDNA copy number was measured by normalizing the expression of NADH dehydrogenase subunit-1 (ND1) gene (mtDNA-encoded) and cystic fibrosis (CF) gene (nuclear DNA-encoded). Primer sequences are listed in Table 1. PCR was carried out with the following settings: denaturation 95 °C for 5 min, followed by 95 °C for 10 s and 63.7 °C for 30 s (40 amplification cycles) in a final volume of 20 μl in 96-well plates (Bio Rad).

### Immunoblot analyses

Whole protein content was isolated from MSC at early and late passages, cultured with or without MB. Cells were lysed in 150 mM NaCl, 1% Triton X-100, 0.5% Na deoxicholate, 0.1% SDS, 50 mM Tris, pH 8.0 (all Applichem) and phenylmethane sulfonyl fluoride (PMFS; Sigma) for 30 min, centrifuged at 800 *g* for 10 min (both at 4 °C), and protein extracts (25 μg) were fractionated by Mini-Protean TGX 4–15% gradient polyacrylamide gels, and semi-dry blotted to a nitrocellulose membrane (both Bio Rad). The nitrocellulose membranes were incubated with mouse monoclonal antibodies against Total OXPHOS Rodent WB Antibody Cocktail (1:1000; ab110413, Abcam), Cu/Zn-SOD (1:2000; rabbit, NBP2-24915SS, Novus Biological), Mn-SOD2 (1:2000; mouse, AM7579A, Abgent, LuBioScience), catalase (1:5000; rabbit, NBP2-24916SS, Novus Biological), and housekeeping gene actin (1:5000; mouse, 3700, Cell Signaling, Bio Concept). Blocked membranes were probed overnight at 4 °C with primary antibodies diluted in 5% milk (Rapilait, Migros, Switzerland) in PBS, followed by HRP-conjugated mouse secondary antibody or rabbit secondary antibody (both 1:10,000; Bethyl, LuBioScience) in 5% milk in PBS for 1 h at room temperature. Membranes were developed with LumiGLO Reserve Chemiluminescent Substrate Kit (LumiGlo Reserve KPL, Bio Concept). Acquisition was performed with a digital SLR camera (Nikon D600, Nikon, Zürich, Switzerland), and the results were normalized to the relative amount of actin.

### MSC in vitro differentiation into osteogenic, chondrogenic, and adipogenic lineages

MSC cultures were stimulated with the appropriate differentiation medium according to the conditions described below.

#### Chondrogenic differentiation

Collagen type I cubes (Biopad, Euroresearch, Italy) were used as a scaffold to support cellular differentiation [31]. MSC (8 × 10^5^) were seeded per cube and kept for 30 min to allow adhesion before the addition of chondrogenic medium. MSC-collagen constructs were cultured for 3 weeks in chondrogenic medium consisting of advanced DMEM + GlutaMAX (Gibco), 2.5% FBS, 100 units/mL penicillin, 100 mg/mL streptomycin, 2.5 μg/mL amphotericin B, 40 ng/mL dexamethasone (Sigma), 50 μg/mL ascorbic acid 2-phosphate (Sigma), 35 μg/mL l-proline (Sigma), 1× Insulin-Transferrin-Selenium X (Gibco), and 10 ng/ml transforming growth factor (TGF)-β1 (Peprotech).

Glycosaminoglycan (GAG) accumulation was determined as a chondrogenic marker. GAG accumulation was quantified with alcian blue binding assay after 6 h digestion of two constructs per sample at 60 °C with 125 μg/mL papain (Sigma-Aldrich) in 5 mM l-cysteine-HCl (Fluka), 5 mM Na-citrate, 150 mM NaCl, and 5 mM EDTA (all AppliChem). GAG accumulation was determined by binding to alcian blue (Fluka, Sigma) and quantified using chondroitin sulphate (Sigma) reference standards [32].

#### Osteogenic differentiation

MSC in monolayer at a density of 5 × 10^3^ cells/cm^2^ were differentiated in STEMPRO® Osteogenesis Differentiation Kit (Gibco) for 3 weeks. Calcium content was determined using the Calcium CPC LiquiColor test kit (StanBio, Schwetzingen, Germany). Cells were washed with PBS, incubated with 0.5 N HCl for 30 min at room temperature and then with O-Cresolphthalein complex one in alkaline solution. Calcium concentration was measured and quantified with standards.

#### Adipogenic differentiation

MSC were cultured in monolayers at a density of 2.5 × 10^4^ cells/cm^2^ under two different culture conditions: adipogenesis inducing medium—basal medium (DMEM/Ham’s F12+ GlutaMAX, 2.5% FBS, 100 units/mL penicillin, 100 mg/mL streptomycin, 2.5 μg/mL amphotericin B) supplemented with 1 μM dexamethasone, 0.5 mM 3-isobutyl-1-methylxanthine, 0.5 mM indomethacin, and 170 mM insulin (all Sigma)—and adipogenesis maintenance medium—basal medium supplemented with 170 mM insulin. Lipid droplets were revealed by staining with Oil Red O (Sigma), and dye content was quantified after isopropanol elution and spectrophotometry by measuring the absorbance at 520 nm.

### Statistical analysis

Results are presented as mean ± SEM. Two tailed Student’s *t* test was used to determine significant differences between samples. For all tests, *p* < 0.05 was considered significant.

## Results

### Cell culture fitness comparison between MSC

**Fig. 1:**
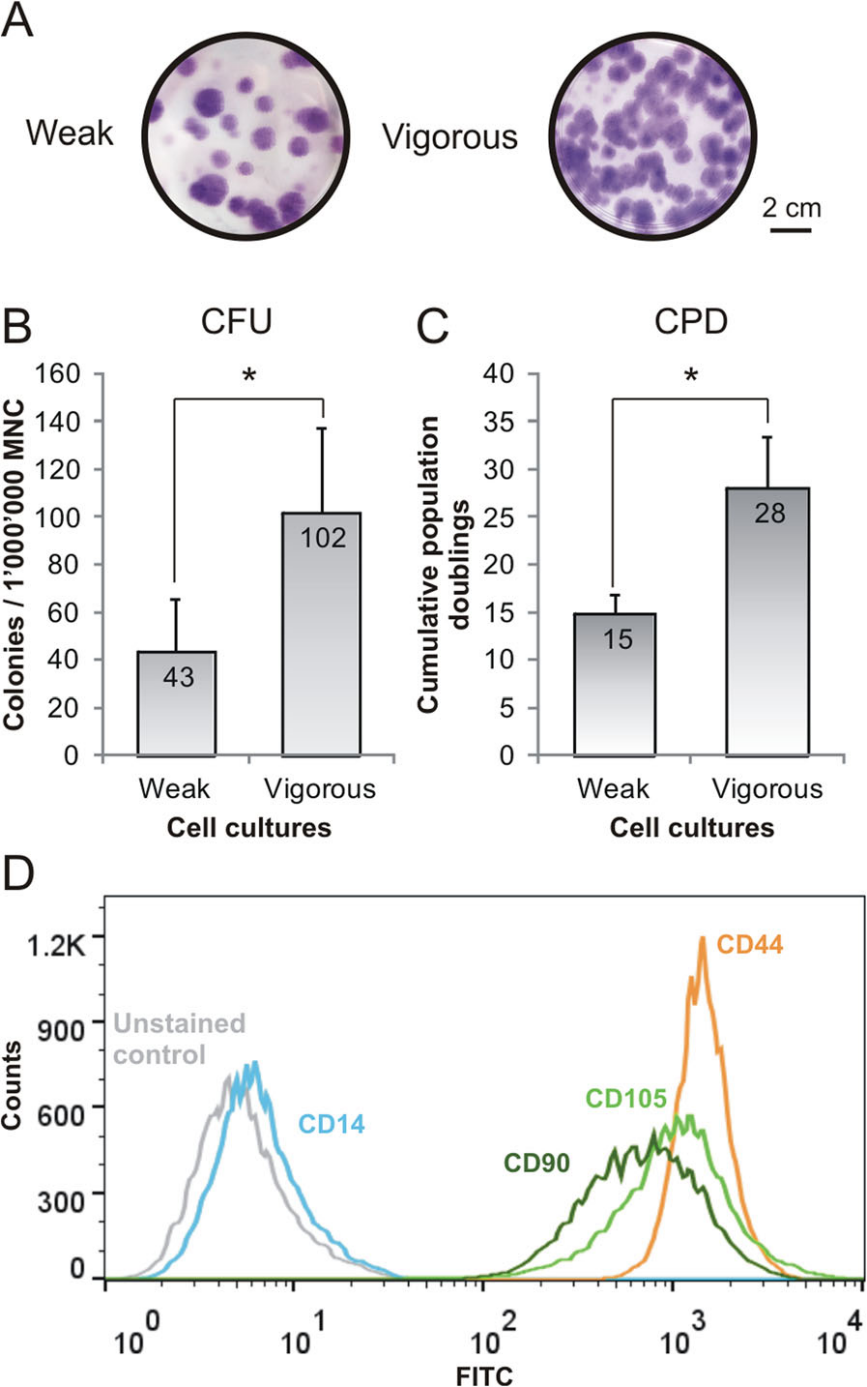
MSC cultures were separated based on their cell fitness in vitro into weak and vigorous cell cultures. **a** Colonies were stained with Giemsa (colony-forming unit assay (*CFU*)) and **b** counted at the beginning of in vitro cultures, while **c** MSC cumulative population doublings (*CPD*) were calculated at the end of cell cultures (*n* = 3; values represent the mean ± SEM). *p < 0.05. Expression of the MSC-positive markers CD44 (*orange line*), CD90 (*dark green line*), and CD105 (*light green line*), and the negative marker CD14 (*blue line*) was tested by flow cytometry analysis (**d**), as shown by a representative sample (*grey line* = unstained control). *MNC* mononuclear cells

MSC in vitro fitness was evaluated based on their ability to form colonies (colony-forming unity assay (CFU); Fig. 1a and b) and cumulative population doublings (CPD; Fig. 1c), and subsequently split in two groups: weak and vigorous. At the beginning of the culture, MSC belonging to the vigorous group formed 2.3-fold more colonies (102 colonies) compared to the weak group (43 colonies; *p* < 0.05). Additionally, CPD were almost double in the vigorous group (28 divisions) in comparison to the weak group (15 divisions; *p* < 0.05). Furthermore, MSC were also characterized by flow cytometry analysis (Fig. 1d) with the positive MSC markers CD44, CD90, and CD105 and with the negative monocyte marker CD14. Compared to early passages, CD90 expression was decreasing by 52% in weak MSC (*p* < 0.05) and by 38% in vigorous MSC at late passages (Table 2), while the other markers were constitutively expressed.

**Table 2:** Flow cytometry analysis of MSC markers at early and late passages

Surface	Weak MSC		Vigorous MSC	
markers	Early	Late	Early	Late
CD14^–^	99.4 ± 0.2	99.6 ± 0.1	99.5 ± 0.4	98.8 ± 0.3
CD44^+^	100.0 ± 0.1	100.0 ± 0.2	99.7 ± 0.5	100.0 ± 0.1
CD90^+^	83.4 ± 15.7	31.0 ± 21.6*	95.2 ± 3.8	57.3 ± 49.3
CD105^+^	98.0 ± 1.7	98.7 ± 1.8	99.0 ± 0.8	99.5 ± 0.2

Values are shown as mean % expression ± SD; *n* = 3 per group

*p < 0.05 compared to early passages

*MSC* mesenchymal stem cells

### Mitochondrial activity of MSC cultures

**Fig. 2:**
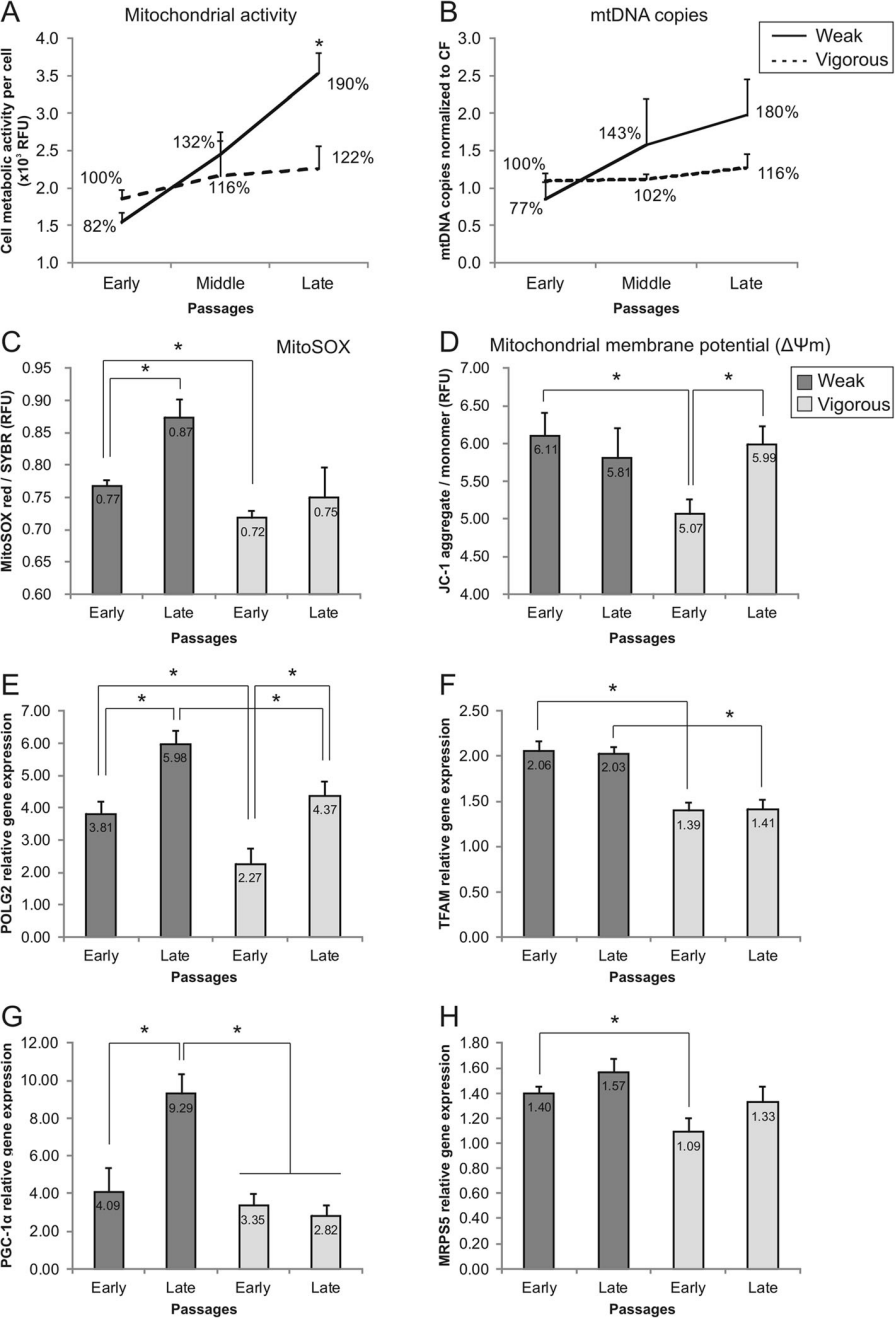
Comparison of cell oxidative status of MSC. **a** Cell mitochondrial activity (measured by resazurin reduction assay), **b** mitochondrial DNA (*mtDNA*) copies (quantified by PCR), **c** mitochondrial superoxide production (*MitoSOX*), and **d** membrane potential (JC-1 staining) were measured in MSC during cell expansion (*n* = 3; values represent the mean ± SEM). *p < 0.05). Gene expression of **e** DNA polymerase gamma-2 (*POLG2*), **f** mitochondrial transcription factor A (*TFAM*), **g** peroxisome proliferator-activated receptor gamma coactivator 1-alpha (*PGC-1α*), and **h** mitochondrial ribosomal protein S5 (*MRPS5*) by MSC was analyzed in all samples at early and late passages (*n* = 3; values represent the mean ± SEM). Gene expression was normalized to GAPDH and normalized to expression in early passages of vigorous MSC. *p < 0.05

Cell metabolic differences between MSC groups were assessed by comparing mitochondrial activity (resazurin oxidative reaction; Fig. 2a), number of mitochondrial DNA copies (mtDNA; Fig. 2b), superoxide radical production (Fig. 2c), and changes in mitochondrial membrane potential (Δψm; Fig. 2d). Cells belonging to the vigorous group showed no significant changes across passages in their mitochondrial activity (22% increase) and mtDNA copies (16% increase). On the other hand, at late passages, cells in the weak group doubled their mitochondrial activity (*p* < 0.05) and mtDNA copies in comparison to early passages. At early passages, superoxide radical (O_2_^–^) production and Δψm were higher (7% and 18%, respectively) in the weak group compared to the vigorous group (*p* < 0.05). At late passages, in the weak group O_2_^–^ production significantly increased further by 13% (*p* < 0.05), while in the vigorous group only by 4%. Δψm of cells in the weak group did not change significantly, while in the vigorous group it significantly increased to reach the levels of the other group (*p* < 0.05).

Quantitative real-time PCR was used to assess the expression of genes linked to mitochondrial homeostasis: DNA polymerase gamma-2 (POLG2; Fig. 2e), mitochondrial transcription factor A (TFAM; Fig. 2f), peroxisome proliferator-activated receptor gamma coactivator 1-alpha (PGC-1α; Fig. 2g), and mitochondrial ribosomal protein S5 (MRPS5; Fig. 2h) at the beginning and at the end of cell culture. At early passages, POLG2, TFAM, and MRPS5 gene expressions were significantly higher (67%, 48%, and 30%, respectively) in cells belonging to the weak group compared to the vigorous group (*p* < 0.05). The TFAM expression difference between the two groups was also preserved at late passages (*p* < 0.05). At late passages, POLG2 expression increased significantly in both groups compared to early passages (*p* < 0.05)—57% in the weak and 93% in the vigorous groups. PGC-1α expression did not differ significantly at early passages between groups, but doubled in the weak group at late passages (*p* < 0.05), while in the vigorous group it did not change. No gene expression differences were found for DNA polymerase gamma (POLG), nuclear respiratory factor 1 (NRF1), and peroxisome proliferator-activated receptor gamma coactivator 1-beta (PGC-1β) (data not shown).

### Differential expansion potential of MSC cultures

**Fig. 3:**
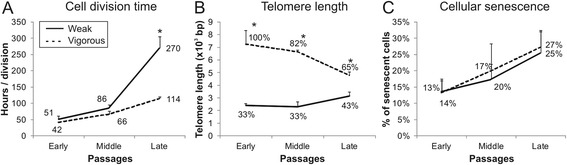
Expansion potential of MSC measured by **a** cell division time, **b** telomere length, and **c** cellular senescence (C_12_FDG intensity measured by FACS) analyzed in weak and vigorous MSC cultures during cell expansion (*n* = 3; values represent the mean ± SEM). *p < 0.05

Expansion potential of MSC cultures was assessed by cell division time (Fig. 3a), telomere length (Fig. 3b), and cell senescence (Fig. 3c). Vigorous MSC cultures were characterized by significantly shorter cell division time in the late passages, compared to weak cells (114 hours/division vs. 270 hours/division, *p* < 0.05). Cells belonging to the vigorous group started with significantly longer telomeres (7200 bp) compared to the other group (2400 bp, *p* < 0.05). This gap was reduced at late passages (4700 bp vigorous versus 3100 bp weak) but was still significantly divergent (*p* < 0.05). Despite these clear-cut differences in cell fitness, the two cell groups showed similar cell senescence patterns across all cell culture passages.

### Methylene blue influence on expansion potential of low oxidative status MSC

**Fig. 4:**
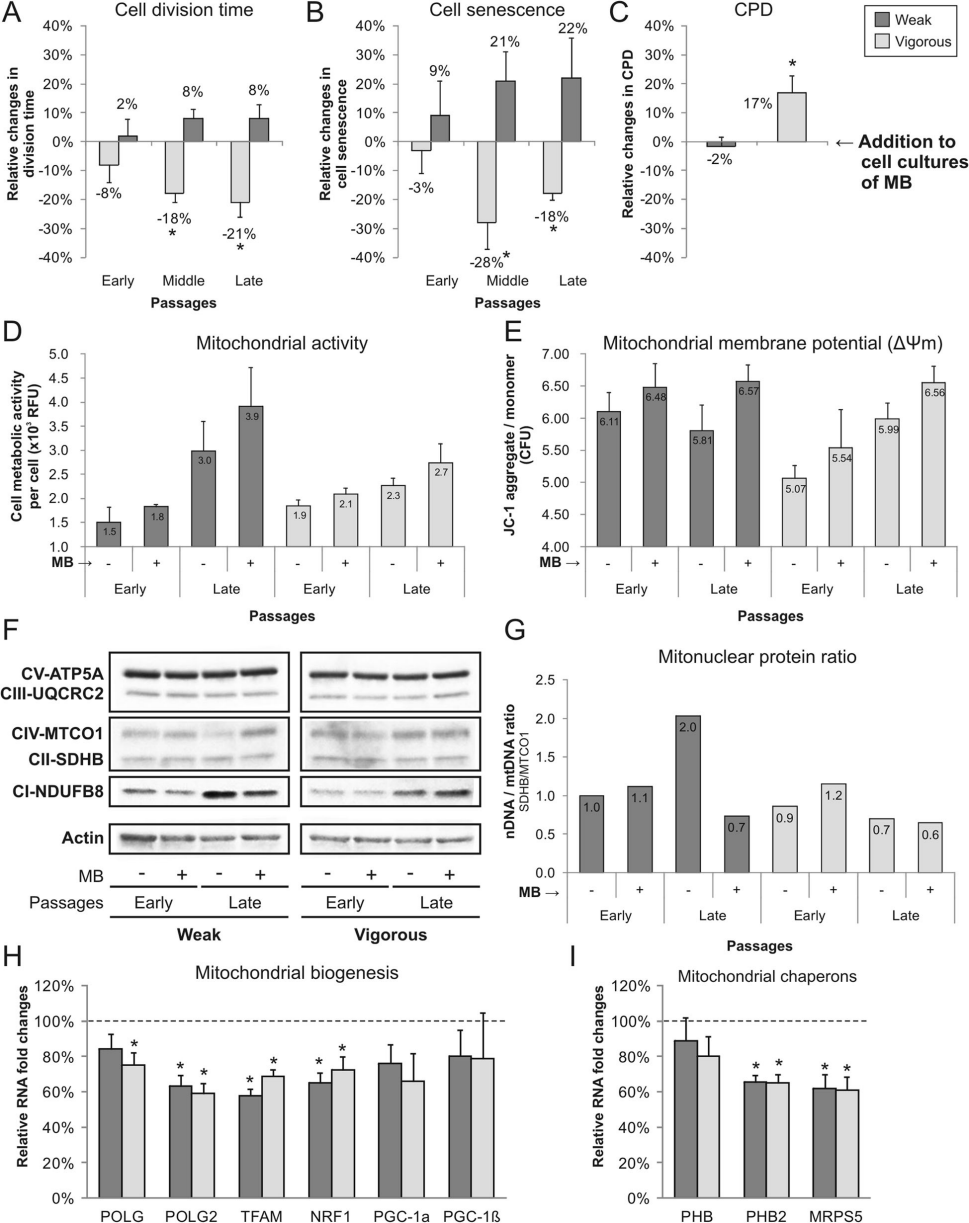
Effects of methylene blue (*MB*) on MSC in vitro cultures. Consequences of MB addition to cell cultures were evaluated by analyzing MSC expansion potential (relative changes were normalized to MSC cultures without MB): **a** cell division time, **b** cellular senescence (C_12_FDG staining), and **c** cumulative population doubling (*CPD*). The effects of MB on cell metabolism were analyzed by **d** cell mitochondrial activity (resazurin reduction assay) and **e** mitochondrial membrane potential (JC-1 staining) (*n* = 3; values represent the mean ± SEM. *p < 0.05. **f** Western blot analysis visualized changes in protein levels of respiratory enzymes in MSC cultured with or without MB, and **g** mitonuclear protein ratio was quantified by dividing SDHB (nuclear-encoded gene) by MTCO1 (mitochondrial-encoded gene). **h** MB induced expression changes in mitochondrial biogenesis and **i** chaperones genes in weak and vigorous MSC cultures at late passages (*n* = 3; values represent the mean ± SEM. Gene expression was normalized to GAPDH and normalized to expression of MSC cultures without MB (*dotted line*). *p < 0.05

MSC were cultured with and without 200 nM methylene blue (MB), an active electron donor/acceptor. With the addition of MB to the cultures, cells in the vigorous group showed reduced division times by 8%, 18% (*p* < 0.05), and 21% (*p* < 0.05) in early, middle, and late passages, respectively (Fig. 4a). MB also reduced by 28% (middle passages) and by 18% (late passages) the number of senescent cells in the population (*p* < 0.05), again only in the vigorous group (Fig. 4b). In the weak group, MB increased the division time (not significantly) by 8%, as well as the number of senescent cells by 22%. These differences were reflected in increased CDP in vigorous MSC by 17% (*p* < 0.05) with MB addition, while no changes were observed in the weak group (Fig. 4c). MB had no influence at any time point on telomere length and mtDNA copies of both MSC groups (data not shown).

Although MB distinctly affected the cell division potential of the MSC groups, the cell mitochondrial activity (Fig. 4d) and Δψm (Fig. 4e) followed the same pattern in both groups. Addition of MB to cultures increased the mitochondrial activity on average by 20% and Δψm by 10%. Western blotting analysis of the relative levels of the five oxidative phosphorylation complexes showed increased expression of NADH:Ubiquinone Oxidoreductase Subunit B8 (CI-NDUFB8) by MSC in the late passages. Furthermore, the ratio between the nuclear-encoded gene succinate dehydrogenase enzyme, subunit B (CII-SDHB), and the mitochondrial-encoded gene cytochrome-c oxidase-1 (CIV-MTCO1) showed a twofold increase in cells of the weak group at late passages, while the levels in the vigorous group were stable (Fig. 4f and g). MB lowered the ratio in the weak group to a level equal to that in early passages, but did not affect cells in the vigorous group. At late passages, MB induced a decrease in the gene expression of genes linked to mitochondrial biogenesis (Fig. 4h) and mitochondrial protein folding (Fig. 4i) in both groups. MSC expression of POLG, POLG2, TFAM, and NRF1 was significantly reduced by 30–40% upon addition of MB (*p* < 0.05). Similarly, mitochondrial chaperones prohibitin-2 (PHB2) and MRPS5 were significantly reduced by 40% (*p* < 0.05). No significant reductions were observed in the expression of PGC-1α, PGC-1β, or prohibitin (PHB).

### Methylene blue reduces superoxide radical production of high oxidative status cells

**Fig. 5:**
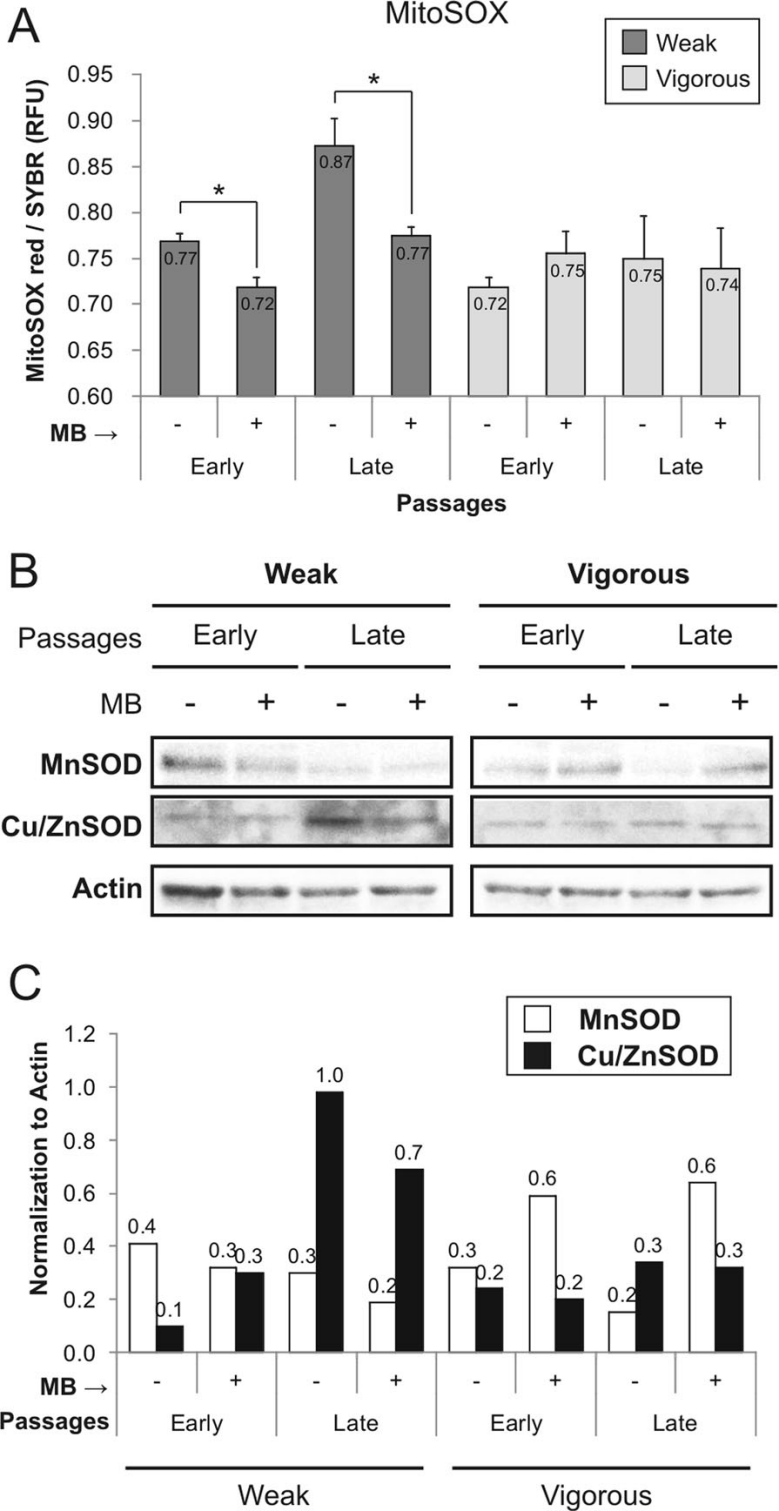
Production of superoxide radicals (O_2_^–^) and expression of antioxidant enzymes during MSC expansion, with or without methylene blue (*MB*) addition. **a** Quantification of superoxide radicals was carried out by MitoSOX staining (*n* = 3; values represent the mean ± SEM). *p < 0.05. **b** Mitochondrial (*MnSOD*) and cytosolic superoxide dismutase (*Cu/ZnSOD*) Western blots were performed on proteins isolated from the same samples, and **c** results were normalized to actin

Higher metabolic rates have been associated with accumulation of toxic reactive oxygen species as a by-product. Measurements of the intracellular levels of O_2_^–^ (Fig. 5a) showed a significant 13% increase in accumulation in late passages in the weak MSC group (*p* < 0.05). MB significantly reduced the production of O_2_^–^ by ~10% (*p* < 0.05). No significant changes in O_2_^–^ production were observed in the vigorous group, either at late passages or with MB. Production of O_2_^–^ shared the same expression pattern of the cytosolic enzyme (copper/zinc) superoxide dismutase (Cu/ZnSOD). In fact, protein levels of Cu/ZnSOD increased at late passages in the weak MSC group, while in the vigorous MSC group they did not (Fig. 5b and c). Protein expression of the mitochondrial enzyme (manganese) superoxide dismutase (MnSOD) was stable across passages, but was more than doubled by MB in the vigorous group. Catalase protein expression was similar between groups and passages (data not shown).

### Methylene blue improves MSC differentiation

**Fig. 6:**
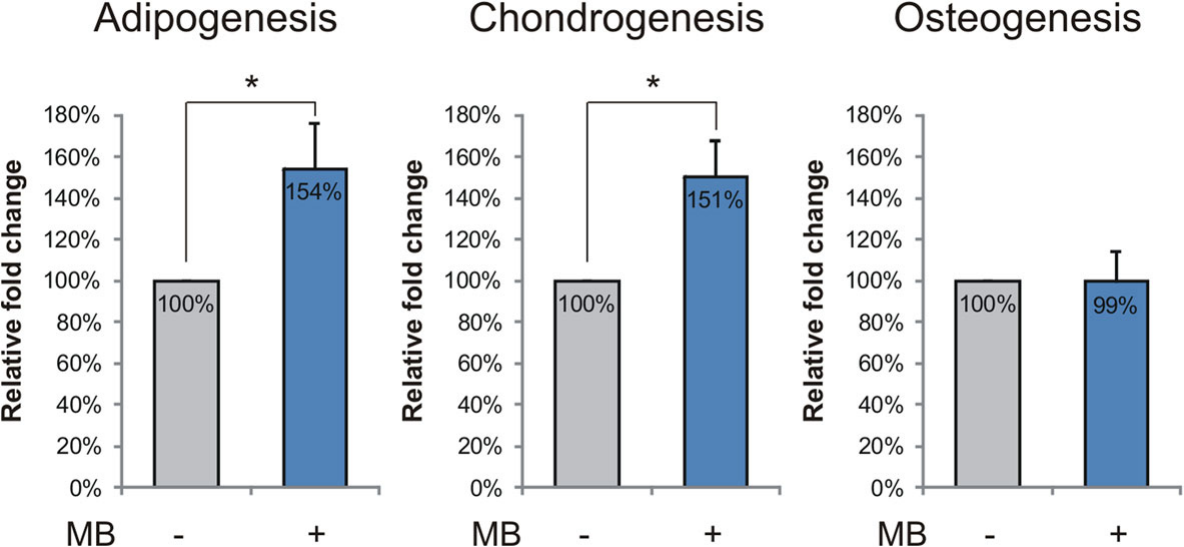
MSC differentiation potential of vigorous MSC at late passages was improved by methylene blue (MB) supplementation to cell culture. MSC were differentiated to adipogenic, chondrogenic, and osteogenic lineages for 21 days, and results were quantified by fat vacuole formation, proteoglycan, and calcium accumulation, respectively (*n* = 3; values represent the mean ± SEM. *p < 0.05

MSC were differentiated to adipogenic, chondrogenic, and osteogenic lineages and results were evaluated by formation of fat deposits and accumulation of proteoglycan and calcium, respectively. MB had no effect on differentiation of weak MSC (data not shown), while it improved differentiation to fat and cartilage by vigorous MSC at late passages (Fig. 6). MB significantly increased adipogenesis and chondrogenesis by approximately 50% (*p* < 0.05), but had no impact on osteogenesis. The differentiation potential between weak and vigorous MSC was similar both at early and late passages (data not shown).

## Discussion

The free radical theory of aging proposes that cumulative damage by free oxygen radicals is the basic reason for cellular and tissue aging. In this study, we showed that MSC isolated from donors of similar age had distinct in vitro expansion potential which mirrored underlying differences in mitochondrial oxidative status.

MSC were divided into two groups, weak and vigorous, based on their fitness in culture. More in-depth analysis demonstrated differences between the mitochondrial function indicators of the MSC: changes in mitochondrial activity, mtDNA copies, superoxide radical (O_2_^–^) production, mitochondrial membrane potential (Δψm), mitonuclear protein imbalance, and expression of transcription factors involved in mitochondrial biogenesis. At early passages, vigorous MSC had lower Δψm and O_2_^–^ production, and expressed lower levels of mitochondrial biogenesis genes, such as POLG2 and TFAM. All these parameters together defined a higher level of cell fitness, further indicated by significantly longer telomeres, reduced loss of CD90^+^ cells, faster division rate, and longer viability in culture. At late passages, vigorous MSC stabilized their metabolic activity at lower levels: fewer mitochondria reflected by fewer mtDNA copies, and consequently decreased production of O_2_^–^. Conversely, weak MSC significantly increased their metabolic activity with culture time, by increasing the number of mitochondria and O_2_^–^, as well as by a rising gene expression of POLG2 and PGC-1α. Although no difference in the progression of replicative senescence was observed between the two MSC groups, these data indicate an energetic dysfunction which affects the long-term lifespan of cells in culture, and shows the limitations of the standard test of replicative senescence when such tests are used as a substitute for cell quality assessment.

Aging of mitochondria is influenced by several maintenance and turnover biological processes. High energetic rates result in accumulation of reactive oxygen species (ROS) which are detrimental for cell components; for instance, mtDNA is very susceptible to oxidative damage [13]. At higher levels, the same is true for high energy consuming tissues, such as the heart and brain, ultimately leading to the formation of damage in tissues and organs [33]. On the other hand, low levels of ROS are well known to trigger an adaptive hormetic response, generating a stress resistant reaction and increased cell longevity [34]. Successful aging has also been related to functional mitochondrial dynamics, where centenarians were shown to have high rates of mitochondrial autophagy [35]. At the molecular level, mitochondrial protein turnover has been associated with extension of longevity by activating the mitochondrial unfolded protein response (UPR^mt^). Mitochondrial misfolded proteins activate chaperones and proteases in order to re-establish the proteostasis balance, promoting a stronger response to stress by the cells [36]. Although a dysfunctional ubiquitin-proteasome system and high rate of protein oxidative damage are among the main causes, UPR^mt^ activation is also elicited by the reduction in the gene expression of mitochondrial ribosomal protein S5 (MRPS5), which is involved in translation of mtDNA-encoded proteins. In fact, 50% downregulation of MRPS5 expression in mice extended their lifespan by ~250 days and, in *C. elegans*, RNA inhibition of the homolog MRPS5 prolonged lifespan by 50% [37]. Also, in our experimental setting, methylene blue (MB) downregulated MRPS5 gene expression by ~40%; however, we could not observe any peculiar UPR^mt^ activation in MSC. Thus, downregulation of MRPS5 alone could not explain the increased MSC lifespan. Additionally, MB reduced gene expression of MRPS5 in the weak MSC without influencing their expansion potential.

MB is a synthetic dye with several interesting features: it is readily available, inexpensive, and approved for medical use. Since its first application as an anti-malarial drug, nowadays MB is used in the treatment of methemoglobinemia [38], cyanide poisoning [39], and ifosfamide-induced encephalopathy [40]. Furthermore, MB enhances mitochondrial oxidative phosphorylation and reduces anabolism in glioblastomas, leading to a decrease in cancer cell proliferation [41]. In August 2016, 86 clinical trials involved MB use, ranging from treatment of pain to bipolar disorder (clinicaltrials.gov). The MB mechanism of action relies on its low redox potential (close to that of oxygen) which allows MB to act as an alternative electron acceptor within the mitochondria. Furthermore, MB has an antioxidant effect, scavenging electrons from the proximity of the enzymes where O_2_^–^ are produced [23]. We also observed an increase in MSC metabolic activity in cultures with MB, coupled with reduced ROS production. Recent observations suggest that an increase in the cell metabolic rate is one of the effects of caloric restriction [42], the only known intervention that extends the lifespan of several organisms.

We speculate that MB promotes the life extension of MSC in a similar way, by promoting increases in cell metabolic rate and mitochondrial membrane potential, and lowering superoxide production. In parallel, we propose that MB preserves functional mitochondria for a longer time due to reduced biogenesis and increased expression of the mitochondrial superoxide dismutase. Similarly, high metabolic rates promoted a lifespan extension in mice, based on the “uncoupling to survive” theory [43]. In mitochondria, protons are transferred to the intramembrane space by the electron transport chain, eventually promoting ADP phosphorylation. However, not all protons contribute to ATP formation, leaking through the inner membrane. This process, known also as mitochondrial uncoupling, supports high cellular respiration and low ROS and ATP production. We also observed that vigorous MSC were characterized by the lowest mitochondrial membrane potential. In a recent study, Sukumar et al. showed that a low mitochondrial membrane potential is a marker of hematopoietic stem cells [44]. Likewise, we propose that MSC with lower mitochondrial membrane potential possess enhanced stemness, which would explain the longer in vitro lifespan. In contrast, in weak MSC, MB could not induce any lifespan extension, although the effects of MB at the molecular level were coherent. We speculate that mitochondria of these cells were in a more advanced state of dysfunction which could not be rescued by MB; hence, MB only has a preventative role.

Limitations of the study were the limited number of donors per group and the absence of more specific tests, such as mitochondrial proton conductance or accumulation of mutations in mtDNA. We included only elderly donors in the project because MSC isolated from older people have lower in vitro expansion potential compared to younger donors [10], and so an increase in cell culture lifespan would be more beneficial for them from a cell therapy prospective. Furthermore, to avoid undesired alterations to the endoplasmic reticulum and mitochondrial properties promoted by the addition of bFGF to the culture media [45], we controlled for this confounding effect by maintaining a constant bFGF concentration among all groups.

## Conclusion

In conclusion, we showed that the oxidative status can be used as a predictor to reliably assess the quality of expansion potential in MSC, independent of a donor’s age. Preserved low oxidative status reduces the oxidative stress in cells, maintaining a prolonged resourceful homeostasis in MSC. We also demonstrated that the addition of MB at a low dose to cell cultures increased in vitro lifespan, but only for those cells with a low starting oxidative status. MB also had the beneficial effect of preserving the differentiation potential in vigorous MSC. Therefore, this study opens the way for further research on the importance of oxidative status in MSC homeostasis, and future applications of MSC in cell-based therapies. Together with our previously published score set for cell senescence [46], analysis of oxidative status might increase the cost-effectiveness and reduce the rate of unsuccessful advanced cell therapies.

## Abbreviations

ADP: Adenosine diphosphate
ATP: Adenosine triphosphate
BM: Bone marrow
C_12_FDG: 5-Dodecanoylaminofluorescein di-β-D-galactopyranoside
CF: Cystic fibrosis gene
CFU: Colony forming unity assay
CII-SDHB: Succinate dehydrogenase enzyme, subunit B
CIV-MTCO1: mitochondrial-encoded gene cytochrome-c oxidase-1
CPD: Cumulative population doublings
FBS: Fetal bovine serum
FGF: Fibroblast growth factor
GAG: Glycosaminoglycan
JC-1: 5,5′,6,6′-Tetrachloro-1,1′,3,3′-tetraethylbenzimidazolylcarbocyanine iodide
MB: Methylene blue
MRPS5: Mitochondrial ribosomal protein S5
MSC: Mesenchymal stem cells
mtDNA: Mitochondrial DNA
ND1: NADH dehydrogenase subunit-1 gene
O_2_^–^: Superoxide radical
PGC-1α: Peroxisome proliferator-activated receptor gamma coactivator 1-alpha
PHB: Mitochondrial chaperones prohibitin
PHB2: Mitochondrial chaperones prohibitin-2
POLG2: DNA polymerase gamma-2
ROS: Reactive oxidative species
SOD: Superoxide dismutase
TFAM: Mitochondrial transcription factor A
TGF: Transforming growth factor
UPR^mt^: Mitochondrial unfolded protein response
Δψm: Mitochondrial membrane potential
